# The contagious nature of imprisonment: an agent-based model to explain racial disparities in incarceration rates

**DOI:** 10.1098/rsif.2014.0409

**Published:** 2014-09-06

**Authors:** Kristian Lum, Samarth Swarup, Stephen Eubank, James Hawdon

**Affiliations:** 1 Network Dynamics and Simulation Science Laboratory, Virginia Bioinformatics Institute, Virginia Tech, Blacksburg, VA, USA; 2 Department of Sociology, Virginia Tech, Blacksburg, VA, USA

**Keywords:** epidemiological criminology, agent-based model, incarceration, susceptible–infected–susceptible model, influence network, simulation

## Abstract

We build an agent-based model of incarceration based on the susceptible–infected–suspectible (SIS) model of infectious disease propagation. Our central hypothesis is that the observed racial disparities in incarceration rates between Black and White Americans can be explained as the result of differential sentencing between the two demographic groups. We demonstrate that if incarceration can be spread through a social influence network, then even relatively small differences in sentencing can result in large disparities in incarceration rates. Controlling for effects of transmissibility, susceptibility and influence network structure, our model reproduces the observed large disparities in incarceration rates given the differences in sentence lengths for White and Black drug offenders in the USA without extensive parameter tuning. We further establish the suitability of the SIS model as applied to incarceration by demonstrating that the observed structural patterns of recidivism are an emergent property of the model. In fact, our model shows a remarkably close correspondence with California incarceration data. This work advances efforts to combine the theories and methods of epidemiology and criminology.

## 1. Introduction

The rapid increase in the US incarceration rate over the last few decades has been described as an epidemic. According to the Bureau of Justice Statistics, the *per capita* rate of incarceration nearly quadrupled between 1978 and 2011, from 137 to 511 persons per 100 000 [1]. This prison boom has primarily affected Black Americans, especially Black males. By 2011, Black incarceration rates were over six times higher than White rates (3023 per 100 000 for Blacks, and 478 per 100 000 for Whites).

Racial disparities in incarceration rates have been studied extensively [2–6], and while these disparities are partially due to differences in criminal involvement [7], the increase in imprisonment for Black males since 1980 was not matched by a similar increase in Black-male criminality [8–10]. What then accounts for the racial disparities in incarceration? Scholars offer several explanations, including differential exposure to police surveillance [9], prosecutorial discrimination [11], the use of incarceration to deal with a ‘racial threat’ [12,13] or sentencing disparities between Blacks and Whites. Although studies reveal that racial sentencing disparities are reduced when legal factors [14–16] or social contexts [17,18] are considered, a recent meta-analysis reports that sentencing disparities remain *even after controlling for these factors* [4]. While the magnitude of the difference is small and variable, it is largest in cases involving discretionary powers and for drug offences. In fact, it has been shown that Blacks receive longer sentences than Whites for drug offences [3,9].

Careful study of patterns of incarceration reveals that incarceration behaves like a contagious disease in that the close associates of an incarcerated person have higher-than-average probabilities of being incarcerated. An individual's incarceration can be ‘transmitted’ to others via several mechanisms. First, an individual's incarceration can increase the family members' emotional and economic stress, and cumulative strains are related to criminal behaviour [19]. Specifically, it has been shown that the children of incarcerated parents tend to display increased levels of behaviour issues [20], including aggressive behaviour [21], a predictor of criminality in later life. Close family members, such as the inmates' domestic partners, experience the acute effects of their family member's incarceration. Decreased household income due to the inmate's inability to work while incarcerated and the inability of the inmate to contribute to child care responsibilities put the remaining family members at increased risk of work–family conflicts, stress and depression [22,23]. Economic losses within the household further percolate to the next generation [24]. Economic and social mobility data show that children born into low income households to parents with low education are themselves more likely to have low levels of educational attainment and to earn a relatively low income in adulthood [25]. These are, of course, risk factors for incarceration, thus reinitiating the incarceration–poverty cycle in a new generation [10,24].

In addition, an individual's incarceration can expose his or her family and friends to a network of criminals, thereby exposing them to criminal norms [26]. These factors may increase the criminality of the incarcerated person's family and friends, thereby increasing the probability that these family members and friends would themselves be incarcerated. Thus, an individual's incarceration can be ‘transmitted’ to others. Alternatively, there is evidence that an individual's incarceration can create an ‘official bias’ against his or her family and friends [27–29]. That is, once a person is incarcerated, the police and the courts pay more attention to the incarcerated person's family and friends, thereby increasing the probability they will be caught, prosecuted and processed by the criminal justice system. Thus, even if criminal behaviour is not transmitted from person to person, a similar pattern emerges because a convicted person's associates will be caught and convicted more frequently than will a non-incarcerated person's associates, even if their criminal behaviours are the same. Regardless of the mechanisms involved, the incarceration of one family member undoubtedly increases the likelihood of other family members being incarcerated [20,21,30,31]. This suggests that models of contagion may aptly characterize incarceration.

It is well known that some models of contagion exhibit nonlinearities. Nonlinear processes such as infectious disease outbreaks are capable of amplifying small differences in parameters through feedback in certain circumstances. One such example is the susceptible–infected–suspectible (SIS) model in which individuals transition between susceptible and infectious states—near a critical value of transmission probability, positive feedbacks amplify small differences in transmission rate to create large differences in *prevalence*, the number of infected people.

It has become popular to use the metaphor of ‘contagion’ for many kinds of social dynamics. The central theme of twentieth century studies of diffusive processes—including contagion—in many disciplines (including theoretical and applied epidemiology, graph theory, physics and computer science) was elucidation of requirements for the existence of a critical point and its properties. This critical point is known as ‘herd immunity’ in epidemiology. Here, we illustrate the implications of this critical behaviour in those social dynamics for which the metaphor of contagion is applicable. In particular, we examine the consequences of critical behaviour that magnifies small differences in transmissibility to large differences in prevalence. We use simulations to carry out a carefully controlled experiment that would be impossible to arrange in the real world. Specifically, we use an extremely parsimonious model to demonstrate that small but significant differences in prison sentences can induce large differences in incarceration rates. Moreover, when we calibrate this model to data on sentencing, we find that the difference in sentence lengths between Blacks and Whites for drug offences in the USA is large enough by itself to explain the observed difference in incarceration rates. We do not claim that incarceration is contagious, nor that this is the only factor determining differences in incarceration rates. Nonetheless, these results are suggestive and important enough to warrant further research in these areas. Furthermore, we expect that similar nonlinear effects will be found in many other contagion-like social systems.

## 2. The susceptible–incarcerated–susceptible model

The hypothesized ‘transmissibility’ of incarceration suggests that an SIS model in which incarceration is modelled as though it were an infectious disease is appropriate. An agent-based simulation of the SIS model requires three main components: a contact network through which individuals stochastically transmit the disease, transmission probabilities that dictate the rate at which agents transmit to each other and a period of infectivity [32]. Network ties represent opportunities for transmission, and the structure of the network through which the disease spreads affects the dynamics of the outbreak. In a disease model, network ties between agents typically denote physical contact or close proximity; in the case of incarceration, they denote the existence of a familial relationship or close friendship, i.e. the existence of a strong influence between the agents.

In the disease modelling paradigm, transmission probabilities may be a function of characteristics of the individual agents (e.g. elderly individuals have a higher probability of contracting a disease) or the relationship between the agents (e.g. transmission rates are higher between parents and small children). In our model, incarcerated people are considered ‘infectious’ to those who are most profoundly affected by their absence. Incarcerated people ‘transmit’ the incarceration ‘disease’ to their network members with a probability that is a function of the relationship type (e.g. an individual's incarceration has greater effect on his or her child than on a friend) and personal characteristics (e.g. males are more susceptible than females). We denote the probability that infected agent *i* transmits to susceptible agent *j* by *p*(*i → j*).

**Figure 1.**
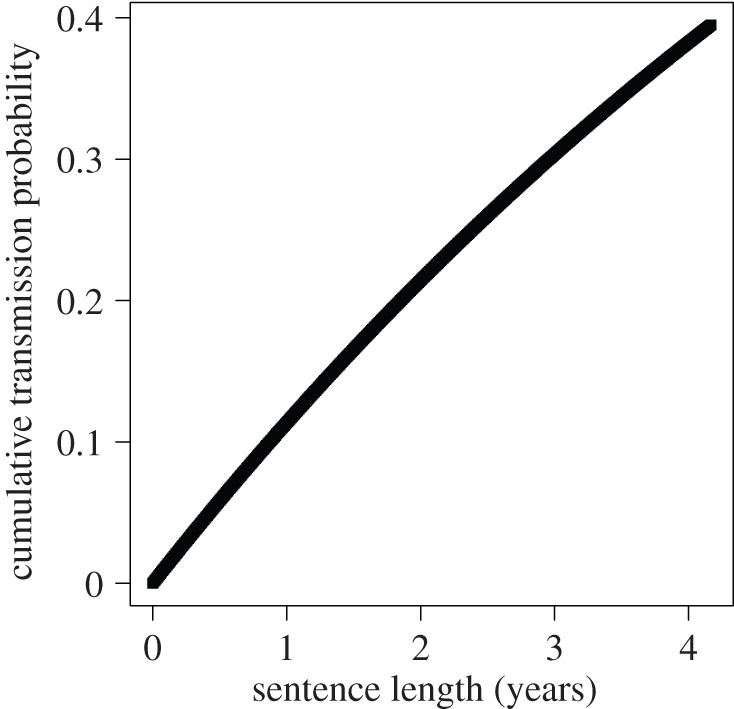
Cumulative transmission probability by sentence length. As an example, we assume that at each iteration (month), an agent transmits with probability *p* = 0.01. This shows the probability that a transmission would have been made throughout the duration of the sentence as a function of sentence length in months.

In modelling the spread of disease using the SIS model, the period of infectivity, *s*, is the duration of time during which the infected individual is contagious. In our model, *s* is the length of the individual's prison sentence, as that is the time during which the inmate's family and close friends are acutely affected. Here, we do not explicitly model increased risk of incarceration due to an inmate's difficulty re-integrating into society. In our model, individuals released from prison cease to be ‘infectious’ and return to a ‘susceptible’ state from which they may become re-infected. The source of the new infection will be incarcerated friends and relations, introducing positive feedback into the system. Given the infectious period, the probability that agent *i* transmits the disease to agent *j* over the whole course of the infectious period is given by 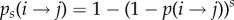, i.e. the complement of the probability that agent *i* does *not* transmit to agent *j* in any of the *s* iterations during which it is infectious. Thus, for fixed *p*(*i → j*), a longer infectious period results in a higher transmission probability (figure 1) and a greater chance that the outbreak becomes widespread.

## 3. Simulation model

### 3.1. Overview

Simulating the described contagious process requires three main components: a synthetic population through which the ‘disease’ will be passed, transmission probabilities and durations of infectivity. In our simulation, we synthesize a realistic multi-generational population of agents for which all family and friendship ties are known. All parameters involved in creating this population are based on recent, high-quality data. For example, distributions for the sex, lifespan and the number of children of each agent are taken from the US Census, the Centers for Disease Control and Prevention and the Social Security Administration, respectively. Transmission probabilities are derived directly from the survey of prison inmates presented in [33]. This survey provides the probability that an inmate's mother, father, sister, brother or adult child are also incarcerated by inmate gender. That is, *p*(*i → j*) is taken directly from the literature. In our simulation, we treat close friends as siblings in terms of transmission probabilities. We focus on the crime of drug possession and use data from the Bureau of Justice Statistics to derive sentence lengths by race. In the following sections, we provide a more detailed discussion of each component of the simulation.

Using this hypothetical synthetic population, we run ‘Black’ simulations in which incarcerated agents are assigned sentences that are consistent with those received by Black Americans for drug possession and ‘White’ simulations using a distribution of sentence lengths corresponding to those received by White Americans for the same crime, as described.^1^ *In order to test whether differential sentencing alone can explain racial disparities in incarceration rates, we use the same transmission probabilities and the same network to represent both Black and White populations*. We acknowledge that there may be differences between the two communities in terms of demographics that affect the structure of the networks (number of children, for example); however, in order to isolate the effect of differential sentencing, we hold all else constant—the network included. Indeed, the ability to isolate one mechanism and disentangle its effect from all others is one of the main advantages of taking a simulation approach.

### 3.2. Synthetic population

#### 3.2.1. Agent attributes

We begin with a seed population of *n* = 1500 individuals, {*a*_1_, *a*_2_, … , *a_n_*}, from which all members of the population will be descended. To initialize each agent, it is assigned several attributes. The *i*th agent, *a_i_*, is assigned a gender from the distribution *g_i_* ∼ Bernoulli(0.5), a birth year (*b_i_* ∼ Uniform(*L*, *U*)) and a spatial location in the unit square 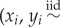 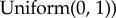. The location may be thought of geographically, as a physical location in a city or as a preference space. Regardless of how one prefers to conceptualize the spatial location, it serves the function of creating communities in the network, as friends and spouses are selected with respect to these locations. To simulate a realistic distribution of life durations, the age at death is sampled according to the 2009 life tables released by the Social Security Administration.^2^ In these tables, the probability of death in the next year at each age (from 0 to 119) is given by sex. We treat these as the probabilities of death at each age throughout the simulation. If individual *i* is female and born in year *b_i_*, we select a life duration, *l_i_*, at random from the distribution given in the female life tables (i.e. 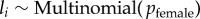). The iteration of death is then given by *d_i_* = *b_i_* + *l_i_*.

Female agents are assigned an age at first birth attribute. Age at first birth is based upon a figure released by the Centers for Disease Control, which lists the mean age at first childbirth for women in 2011 as 25.6 years.^3^ We specify *h_i_*, the age at first birth, to be drawn from the distribution *h_i_* = 15 + *r_i_*, where 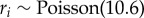, which has mean 25.6.

Each female agent is also assigned the number of children she will have throughout her lifetime. The US Census provides the distribution of the number of children that women in the age bracket 40–44 have had and lists the total fertility rate for several years between 1980 and 2008, which ranges from about 1.84 to 2.1. To prevent our simulated population from dying out (fertility rate < 2), we adjust the raw distribution given for 40- to 44-year-old women to be consistent with historical fertility rates. Under this adjusted distribution, the expected fertility rate is 2.07 children per woman.

#### 3.2.2. Network ties

In addition to personal characteristics, each agent is endowed with relationships with other agents. These are represented as edges in a network. In our simulation, all parent–child, sibling, spouse and close friend relationships are represented. When an agent reaches its 10th ‘birthday’, the agent forms friendship ties. In order to select the number of close friends assigned to each agent, we use data from the 2004 General Social Survey,^4^ in which respondents indicate the number of individuals with whom they discuss important matters and their relationship to up to five of these people. Because many familial relations are already accounted for in our simulation, we count only those people listed who are not parents, children, spouses or siblings to calculate the probability of selecting each possible number of friends, *f_i_*.

Conditional on *f_i_*, we select the specific agents that will be designated as friends. We consider potential friends to be non-siblings between the ages of 9 and 11. This age range is based on information obtained from the National Longitudinal Study of Adolescent Health,^5^ in which children were asked to list several of their friends. Of the friends listed, nearly 95% were within one grade of the student (75% shared the same grade). Because friends within 1 year of the students made up the majority of close childhood friendships, we also restrict to this range. From this set of potential friends, we select the *f_i_* agents that are closest in Euclidean space to the given agent.

In the iteration in which a female actor's first child is born, she is assigned a partner. In this hypothetical population, we only model opposite-sex spousal ties. The algorithm first finds all potential partners—unrelated and non-friend male agents whose current age is between the female agent's age and 9 years older. We selected this scheme based upon data from the 1999 US Census that shows that for about 80% of marriages, the husband's age minus the wife's age falls into the range [−1, 9]. We restrict male agents to be strictly older than female agents, as our partner selection algorithm tends to force the age of the male partners to fall to the lower end of the allowable range. From this collection of potential partners, the agent is assigned the potential partner that is located the closest to it, again using Euclidean distance.

At this point, the children of the couple (or single mother) are initialized. The first child is assigned to be born in the current iteration. Subsequent children are assigned birth years as the current iteration plus independent and identically distributed draws from a Poisson distribution with mean parameter *λ* = 4.5. The mean parameter, *λ*, is again based on the Centers for Disease Control's data used to calibrate the age at first birth. Child locations are set to be half-way between the mother's and father's location plus random noise (Uniform(*m*_1_ − 0.05, *m*_2_ + 0.05)), where *m*_1_ and *m*_2_ are the midpoints between the mother's and father's location along the *x-* and *y-*axis, respectively.

**Figure 2.**
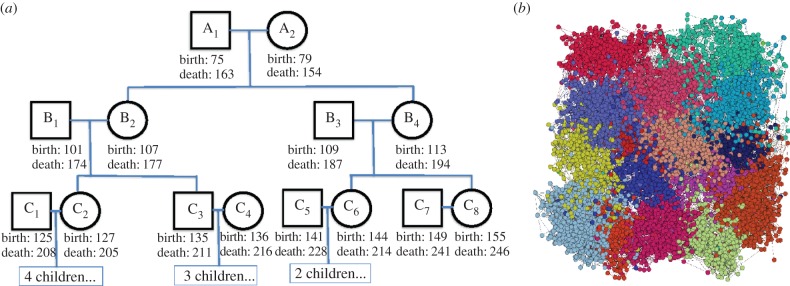
(*a*) One family from the synthetic population. (*b*) The network of agents in our simulation. Different colours indicate communities found in the network. These communities are not explicitly included in the simulation

We run our algorithm for 200 iterations, resulting in a total population of 13 826 individuals. We discard the first 150 iterations as a burn-in period, reducing dependence on our initial conditions. Those agents that are part of our simulation (i.e. those that are alive at any point beyond the 150th iteration) are retained. The total population consists of 8856 agents, with 61 376 family and friendship ties. This total population size is not pre-specified, as it is the result of the random birth and death process initialized with 1500 agents. Figure 2*a* shows an example family tree generated by our algorithm. This individual family, of course, does not exist in isolation of the rest of the population. The population-wide network is shown in figure 2*b*.

### 3.3. Generating sentences

**Figure 3.**
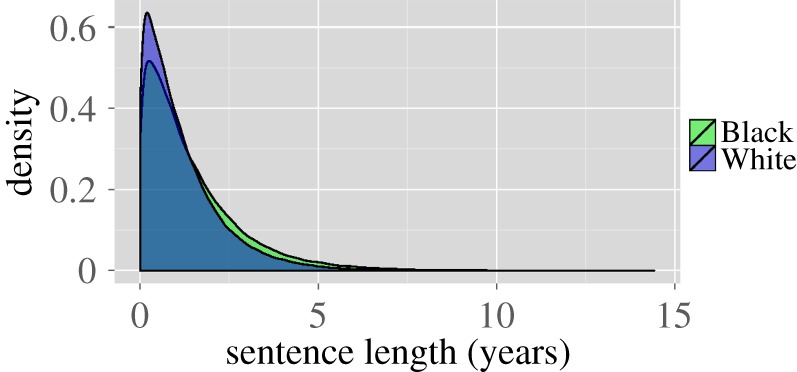
Comparison of the distribution of sentence lengths for Whites and Blacks under our simulation parameters. These distributions are fitted to data released by the Bureau of Justice Statistics.

Data released by the Bureau of Justice Statistics^6^ indicate that the duration of sentence served for the same crime varies by race. In particular, the Bureau of Justice Statistics states that for drug possession, the mean sentence for Whites is 14 months with a median of 10 months. For Blacks, the mean sentence served is 17 months with a median of 12 months. We use a negative binomial distribution to generate sentence lengths that are consistent with the specified summary statistics. A comparison of the sentence distributions is shown in figure 3.

### 3.4. Transmission probabilities

Dallaire [33] presents the results of a survey of incarcerated individuals. In this survey, each inmate is asked which of their relations are also incarcerated. The proportion of inmates whose relations are incarcerated are reported in these data by sex. We use these probabilities to derive our transmission probabilities, *p*(*i → j*). We have noted that if agent *i* has probability *p*(*i → j*) of infecting agent *j* each month it is incarcerated and its sentence is *s* months, then the probability of transmission over the course of its incarceration, *p*_sentence_(*i → j*), is given by *p*_sentence_(*i → j*) = 1 − (1 − *p*(*i → j*))*^s^*. One can easily solve for *p*(*i → j*) = 1 − (1 − *p*_sentence_(*i → j*))^1/*s*^. We set *s* = 14 to approximately calibrate to a value between the Blacks and Whites. The derived monthly transmission probabilities used in this simulation are given in table 1.

**Table 1.** Derived monthly transmission probabilities, *p*(*i* → *j*).

	women	men
mother	0.001	0.003
father	0.011	0.011
sister	0.008	0.004
brother	0.033	0.030
spouse	0.004	0.001
adult child	0.017	0.006

The derived monthly transmission probabilities are most usefully understood in the context of the probability of transmission averaging over sentence length, i.e. the marginal transmission probabilities,3.1
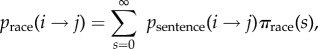
where *p*_sentence_ is defined above and *π*_race_(*s*) is the distribution of sentence lengths for each race. Marginal probabilities by race, sex of inmate and relation are given in table 2, along with the original probabilities listed in [33]. These were calculated using the Monte Carlo method. We note that our marginal probabilities for the White sentences tend to be just slightly lower than those given. For Black sentences, the marginal probability of transmission tends to be slightly higher than that given. This is, of course, unsurprising as the sentences tend to be shorter for Whites and they thus have fewer opportunities for transmission. Recall that the monthly transmission rates under the two scenarios are precisely the same, so one should not interpret the differences in marginal probabilities to mean that this model implies that Blacks are more susceptible than Whites under the same conditions. The only differences that exist here are due to the discrepancies in sentencing. We find that the probability of transmission for Blacks under these parameters tends to be about 20% greater than the probability of transmission for Whites.

**Table 2.** Probabilities given in [33] and marginal transmission probabilities for Whites and Blacks.

	survey		White		Black	
	women	men	women	men	women	men
mother	0.012	0.048	0.012	0.046	0.014	0.056
father	0.147	0.148	0.138	0.138	0.163	0.163
sister	0.107	0.059	0.101	0.058	0.121	0.069
brother	0.377	0.349	0.324	0.303	0.370	0.347
spouse	0.059	0.011	0.057	0.011	0.069	0.013
adult child	0.213	0.085	0.194	0.082	0.227	0.098

The assumption that one's infectivity is constant over the duration of the sentence and non-existent upon release is likely flawed. In reality, the degree of infectivity likely varies over the course of the sentence and slowly decays after releases. Ex-inmates may even have heightened infectivity immediately after release, particularly as they try to reintegrate into their families and communities and find work. However, we find it plausible that the duration of infectivity is at the very least positively correlated with sentence length. If we were to assume that the infectious period were proportional to the sentence length and calibrate the transmission probabilities to this increased period of infectivity, the eventual results of our simulation would be quite similar, as the marginal probability of transmission over the course of the infectious period would be similar. Parsimony is an important quality in an agent-based model, and we have endeavoured to adhere to this principle whenever possible, particularly in cases where there are no data to inform more detailed parameters. In this case, we have no information about the duration and rate of post-release reacclimatization. Thus, we assume that infections only take place during the sentence, a period about which we have concrete data.

## 4. Results

**Figure 4.**
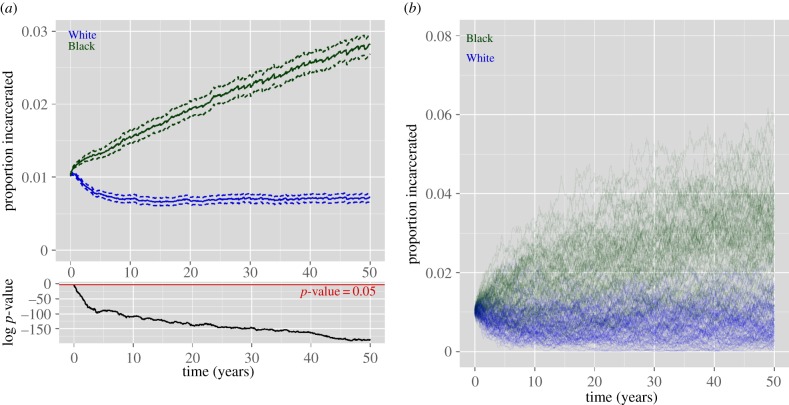
(*a*) The mean incarceration prevalence over time by race. Log *p*-values shown below indicate that at all but the first time point, the mean prevalence is significantly different between the two populations. (*b*) Several example epidemic curves.

We run our simulation 250 times using each sentence-length distribution, resulting in 250 ‘Black’ epidemics and 250 ‘White’ epidemics. The populations are initialized to have approximately 1% of individuals incarcerated at the beginning of the simulation. Although White and Black incarceration rates, in reality, have been disparate throughout history, we initialize the simulations equivalently under the two scenarios to rule out the possibility that the resultant disparities are due to initial conditions alone. Our first analysis looks at the effects of differential sentencing after 50 years, a duration slightly longer than the time since the term ‘War on Drugs’ was coined in the USA. Figure 4*a* shows the mean epidemic curves and corresponding confidence intervals by race; figure 4*b* shows several example trajectories. While the prison epidemic takes off under Black sentence lengths, reaching just under 3% incarcerated on average after the 50 years in our simulation, under the model using White sentence lengths, the prison population first declines and then increases at a much slower rate, reaching about 0.725% over the same time period. Eventually, the Black and White simulations level off (or increase at a negligibly slow rate) at around 7% and 1%, respectively.

**Figure 5.**
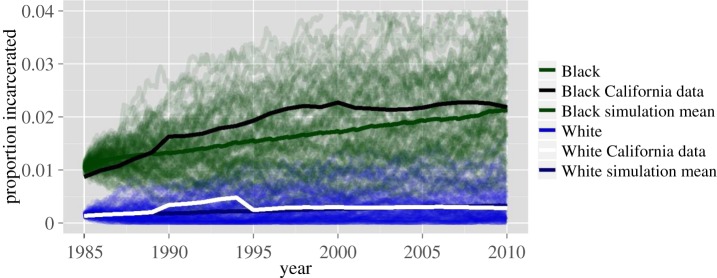
Simulation results and real data. The incarceration rate in California by year and race. The number of incarcerated people by race and year was calculated using the National Prisoner Statistics dataset from the Inter-University Consortium for Political and Social Research. The total number of people in California by race and year was calculated from the California Department of Finance.

We have shown that under a largely arbitrary simulation period and initial values, large racial disparities in incarceration rates are an emergent property of this model. We now test whether this conclusion holds under realistic time periods and initial values. As a comparison with real data, we initialize the Black and White simulations to be near the real incarceration rates in California in the mid-1980s when mandatory drug sentencing became law there (1% and 0.15% for Blacks and Whites, respectively). The results of this are shown in figure 5. From 1986 to 2010, the incarceration rate for Blacks in California climbed from about 1 to 2.18%, whereas the rate for Whites rose much more modestly from 0.15 to 0.277%. In our simulations, the Black and White simulations increase to 2.13% and 0.33%, respectively, over the same time period. For the Black trajectory, our mean trajectory deviates slightly though the ultimate results after 25 years are remarkably similar to reality; however, we note that the real trajectory in California is within the range of values that are typical under our simulation, as it falls well within the cloud of trajectories shown. The White trajectory in California is quite similar to the mean trajectory in our simulation.

Agent-based models are often evaluated with respect to the model's ability to reproduce a set of ‘stylized facts’ as opposed to specific values [34,35]. These facts are often structural or qualitative in nature; examples include patterns of persistence or high autocorrelation in financial time-series [34], spatial clustering of crime [36] or habitation sites [37] and racial segregation in housing [38]. Thus, it is not strictly necessary that a generative model be able to perfectly match observed statistics to establish the plausibility of the generative mechanism; rather, it is necessary that the agent-based model be able to reproduce observed structural properties at a qualitative level. For example, in Schelling's foundational work in describing a generative mechanism for racial segregation in housing, it is enough to show that simple decision rules produce clusters of like agents [38]; one need not establish an exact correspondence between clustering coefficients in these simulations and those seen in reality.

**Figure 6.**
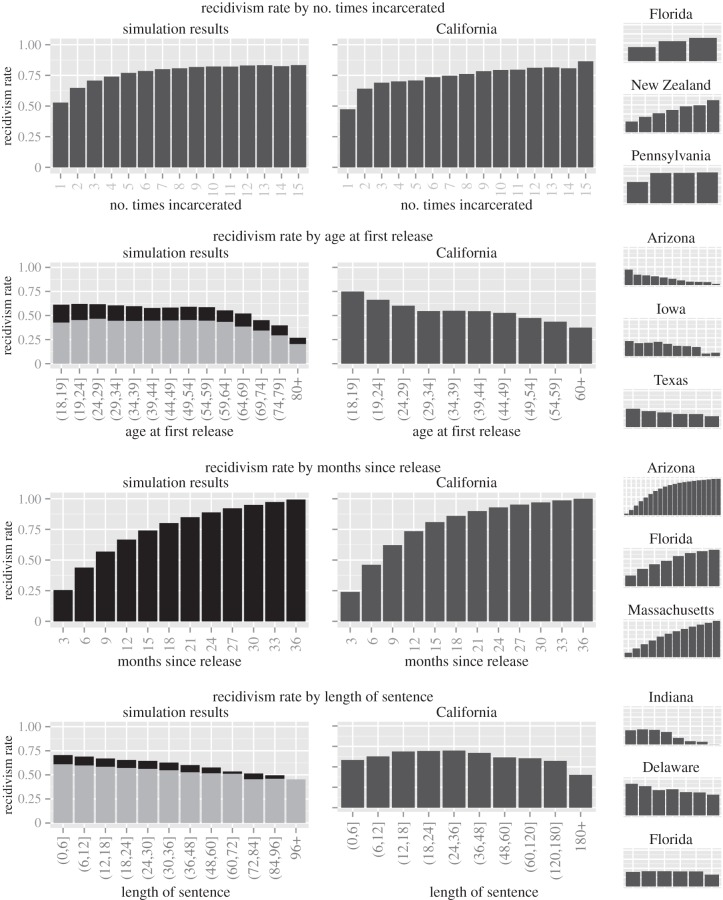
A comparison of the output of our simulation model with recidivism rates in California and several other states. Results from ‘Black’ simulations are shown with black bars, and those from the ‘White’ simulations are shown with lighter grey bars. In the case where differences between the results from the two sets of simulations are visually indistinguishable, we present only the ‘Black’ results.

In that spirit, here we establish the ability of our model to reproduce structural properties of the incarceration epidemic with respect to the patterns found in recidivism data. In figure 6, we compare the recidivism rates derived from the simulation model to those released by the state of California. The plots to the right show the same statistics from a variety of other states. The chosen states vary because not all states release the same statistics; we chose California as our primary point of comparison because its report shows recidivism rates disaggregated by the most factors and at the highest level of discretization. Remarkably, our model reproduces the structural properties of recidivism very accurately. For example, in both our model and the data from California, recidivism rates increase with the number of times an inmate has been incarcerated, with the largest increase occurring between the first and second incarceration. This effect emerges as a structural property of the SIS model *without including an increased probability of incarceration for those who have previously been incarcerated*. An agent's incarceration affects its local network enough that, upon release, it has a higher probability of return. In all cases, including our simulation results, the recidivism rate is lower for those who are released at an advanced age. In fact, our simulation even reproduces a subtle demographic bulge in rates that is apparent in the California report. This is again an emergent property of the model—as an agent ages, it tends to have fewer (and different types of) contacts who generate positive feedback for incarceration. The rate by months since release (for those who recidivated within 3 years) closely matches the structure of the data from all states. These results suggest that our model is reflecting the underlying regularities in the system.

**Figure 7.**
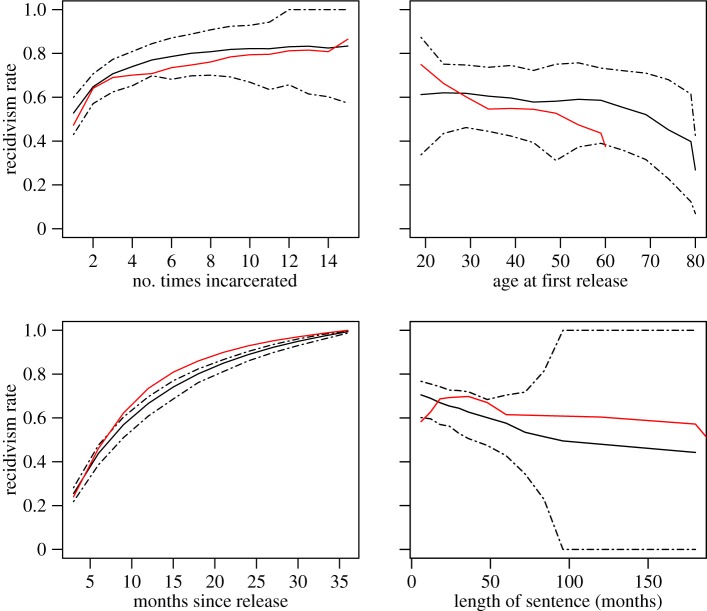
Simulation intervals for the recidivism statistics. The dashed lines show the 97.5th percentile and the 2.5th percentile of the statistics calculated over 250 simulations. The mean over the simulations is shown by the black line and the values in the California data are shown in red.

In addition to reproducing the structural properties of incarceration at a qualitative level, the contagious model of incarceration also shows a very close correspondence to the actual recidivism rates, adding additional weight to the plausibility of the contagious theory of incarceration. Figure 7 shows the recidivism statistics with 95% simulation intervals for the ‘Black’ simulations; the results of the ‘White’ simulations are very similar, and for the sake of clarity, are omitted. The dashed lines show the 97.5th percentile and 2.5th percentile of the statistics over the 250 simulations. The mean across simulations is shown by the solid black line; the values reported in the California data are shown in red. For the most part, in addition to reflecting the *underlying structure* in the patterns of recidivism, our model also matches quite closely with the recidivism rates themselves. In most cases, the actual values fall within the range of our simulation. We caution that the width of the simulation intervals are almost entirely a function of the number of agents in the simulation model and for a larger simulated ‘town’, these intervals may be much smaller. More standard confidence intervals (not shown) as opposed to simulation intervals should similarly be interpreted with caution, as they can be made to be arbitrarily small simply by increasing the number of simulations run.

These plots do reveal some notable differences between the results of our simulation and the real data. First, the trend in recidivism by age appears to be a ‘stretched out’ version of the real trend. This discrepancy is likely a function of the fact that the agents in our simulation tend to live quite long—probably much longer than the real population of individuals that tend to get incarcerated. Incorporating dependence between an agent's incarceration and its lifespan would likely reduce this discrepancy. Second, our simulation tends to miss the increased risk of recidivism for young people. Age-specific transmission probabilities could be tuned to close this gap. Finally, we note that the length of sentence error bars become quite wide past about 8 years. This is because few sentences in each simulation fall in this range, and thus the values in some simulations may be based upon very few data. Simulations with more agents would undoubtedly reduce the width of these bars, as there would be more samples from the tails with which to calculate the proportions in each simulation. Regardless, the mean across all of the simulations (black line) is still an unbiased estimate of the overall rate of recidivism for long sentences under our model and reflects the trend as a function of sentence length.

The discrepancies between the real data and the simulated data offer suggestions for refinements to the model that could improve its accuracy. Such refinements, however, come at the expense of parsimony. Given how well our extremely parsimonious model is able to reproduce many facets of the incarceration epidemic, we anticipate that the marginal returns for incorporating the described refinements will be negligible. *That is, although we may be able to more precisely match exact figures from the real data with a more complicated and highly parametrized model, realizing small improvements to the accuracy of the incarceration and recidivism rates in our simulation will do little to further establish the plausibility of our hypotheses. In its current form, our model clearly demonstrates the plausibility of our hypothesis that incarceration may be spread in a manner similar to a contagious disease and that sentencing disparities may be a major cause of racial differences in incarceration rates.*

### 4.1. A non-contagious model

**Figure 8.**
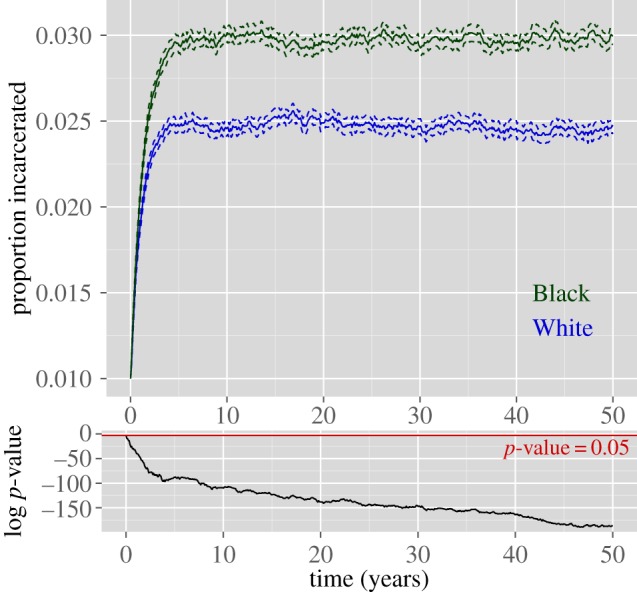
Proportion of people incarcerated by time under the non-contagious model.

It is important to test whether the causal mechanism of interest (in this case, contagion) is a necessary component of the model [36,37]. If it is possible to reproduce an equivalent fit to the structural properties of the observed process without the mechanism, then, under an appeal to parsimony, it is not sensible to put forth the mechanism as a plausible candidate for the true causal mechanism. We address this issue by running a simulation in which incarceration is not contagious. In these simulations, the mechanism of interest is ‘turned off’. Here, there is only spontaneous infection—at each iteration, each agent has equal probability, *p*, of becoming infected independent of any relationship with an incarcerated agent. In order to make a fair comparison, in this simulation we tune the probability of spontaneous infection such that, at the end of the simulation, the Black population's prevalence is roughly 3% as in the SIS simulations. We again initialize both populations to have a 1% incarceration rate at the outset and use the appropriate sentence-length distributions for Black and White simulations. Figure 8 shows the results of this experiment. Under this model, we are not able to reproduce the large difference in incarceration rates between the Black and White simulations. Here, the curves are only about 20% different, whereas in the simulations that incorporate the contagious mechanism, as well as in reality, the incarceration rate of Black agents is many times that of the White agents. The trajectory of the epidemic under this scenario also fails to resemble that of the real incarceration epidemic—it takes off very quickly and within approximately 5 years reaches equilibrium (figure 8).

**Figure 9.**
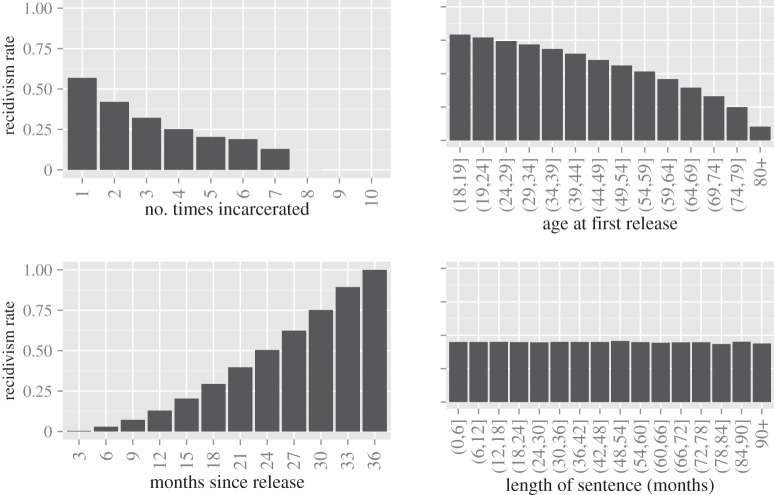
Non-contagious simulation results. Recidivism trends under the non-contagious model.

Recidivism statistics that pertain to the non-contagious simulation appear in figure 9. Under the non-contagious model, we find a very poor correspondence to reality. Under this model, the rate of recidivism with respect to the number of times previously incarcerated is decreasing, whereas in the contagious simulation and the real data it is increasing. Here, the cumulative rate of recidivism by months since release shows a convex pattern where both data generated by the contagious model as well as the real data exhibit a concave shape. The probability of recidivism is essentially flat with respect to the length of the sentence under a non-contagious model; real data and the contagious model both show that longer sentences result in a decreased probability of recidivism. Recidivism by age is the only metric by which the non-contagious model roughly reproduces the same results as seen in the real data, though even here the shape of the curve appears markedly rounder than in the California data and lacks the characteristic demographic bulge.

Based on the lack of correspondence between reality and recidivism data generated under a non-contagious model coupled with the close correspondence between reality and data generated by the contagious model, we conclude that sentence-length disparities in the absence of network effects do not account for the observed difference in incarceration rates. It is feedback through the local network coupled with sentencing disparities that causes such large differences in incarceration rates.

### 4.2. An ordinary differential equations approach

Under assumptions of random mixing and homogeneity of transmission rate, the SIS model can be written as a set of ordinary differential equations (ODEs). In this case, the number of people infected—the *prevalence*—when an outbreak reaches a steady state, *I*, is determined by the expected number of transmissions per infected person, which in turn is given by the product *ps* of transmission rate and the duration of infectivity. The presence of a positive feedback loop makes the relation between prevalence and number of transmissions highly nonlinear. In particular, ignoring births and deaths, *I* = 0 if *s* < *p^−^*^1^ and 1 − 1/*ps* otherwise. Thus, given the transmission rate *p*, we can define a critical duration of infectivity 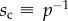 such that for *s* < *s*_c_ the outbreak dies out, while for *s* > *s*_c_ it achieves a non-zero steady-state prevalence. Near *s*_c_, small differences in sentencing (i.e. duration of infectivity) can cause large differences in incarceration (i.e. disease) prevalence.

We take an agent-based simulation approach to modelling the incarceration epidemic because neither the assumption of uniform mixing nor the assumption of homogeneity is met. Indeed, the network of family and friends plays a *crucial* role in our hypothesis. Furthermore, the data show that transmission rates depend on the nature of the relationship between the infectious and susceptible people, and any particular susceptible may simultaneously have several types of relationships with different infectious people (e.g. mother *and* sister *and* daughter). It is easier to capture this heterogeneity in transmission rates in an agent-based simulation than in a set of ODEs.

**Figure 10.**
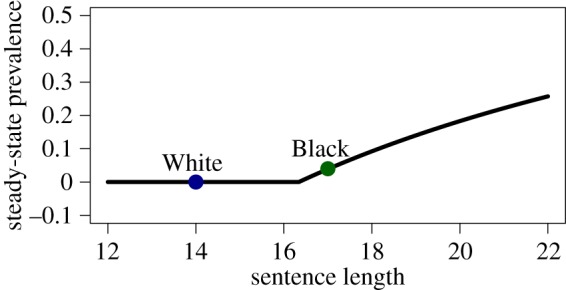
Steady state of ODE model. Under a simplified ODE model of transmission, the steady-state prevalence is given by the black curve as a function of sentence length and, implicitly, transmissions per infected person per month, which is estimated from the agent-based model. The mean sentence lengths of both Black and White populations are indicated on the plot, showing that the mean sentence lengths lie on opposite sides of the bifurcation.

Moreover, using the output of our simulation, we can *generate* a population-wide mean transmission rate *p* to calibrate an ODE model. The result is *p* ≈ 0.0612 transmissions per infected person per month, or *s*_c_ ≈ 16.3 months. Thus, the mean sentence lengths (17 and 14) are on opposite sides of the critical point, illustrated in figure 10. Under this model, the incarceration epidemic in the White population would eventually die out. However, the Black population's incarceration rate would reach a steady state of about 3.9%. As this model does not account for spontaneous infections, this result is consistent with those of the agent-based model. This approach, however, does not allow us to assess the validity of the model or comment on its ability to reproduce structural properties of the epidemic using other withheld sources of information, such as the recidivism data we have shown.

## 5. Future directions

Incarceration as a contagion is a complex process that co-evolves with many other complex social processes, e.g. poverty. Our model by no means accounts for all of the complexities in the process; however, we view the simplicity of our model as one of its major strengths. Even without accounting for the details, our model is able to capture many of the facets (or ‘stylized facts’) observed in real incarceration data.

Extensions to this model could nonetheless prove fruitful. For example, one could investigate the extent to which differences in network structure between Black and White communities might encourage or discourage the spread of incarceration. In particular, increasing the fertility rate in certain communities would raise the average degree in the network, giving more opportunities for transmission. One might investigate whether the differences between Black and White communities in fertility rate plausibly play a significant role in the spread of incarceration. Black and White communities do not exist completely separately; real networks include both Black and White individuals. One might simulate a more realistic mixed-race influence network to investigate the extent to which the rate with which the two communities are commingled might affect the epidemic, even under inequitable sentencing for Blacks. In this work, we have ignored the residual effects of incarceration once the agent is released. Future work might seek to model an extended infectious period during which the ex-inmate is trying to reintegrate into their home life and find work. Last, one very interesting extension would be to simulate the mechanisms by which incarceration is spread. People with low income are more likely to become incarcerated, and incarceration decreases one's expected lifetime earnings. These effects are passed on through the generations, as social mobility data indicate the children of low income parents are themselves likely to have low income, and thus are at increased risk of incarceration. It has also been documented that incarceration increases the likelihood of relationship dissolution and prevents young men from forming stable long-term relationships. A lack of stable family life is a risk factor for incarceration, increasing the likelihood of re-incarceration for the ex-inmates as well as for their children. Understanding and quantifying the relationship between poverty and incarceration could have a potentially large impact, as the insights gained could be used to help drive policy decisions.

## 6. Discussion

The model presented here demonstrates that the dramatic disparities in incarceration rates of Black and White Americans can be generated by the ‘transmission’ of incarceration from an incarcerated person to his or her family and close friends combined with modest differences in sentencing. A relatively small difference in sentencing of, on average, three months over a period of approximately 25 years created incarceration discrepancies similar to those observed today. The plausibility of our model is further supported by its agreement with observed patterns of recidivism, especially in California. However, the model reveals that, contrary to the arguments of some advocates, sentencing differences alone are unlikely to account for the disparities. To generate the large incarceration disparities observed today in the USA, the model must include both sentencing disparities and a mechanism for transmitting incarceration through social networks. Our model does not seek to address why disparities in sentencing exist. Rather, it demonstrates that disparities in incarceration rates between White and Black Americans may have as much to do with social influence as the criminal behaviour of individuals.

More broadly, we have demonstrated that a model of contagion produces an incarceration epidemic similar to that observed in the USA regardless of the demographic characteristics of the individuals. If incarceration risk is indeed propagated through social networks, our results predict that incarceration is self-perpetuating and changes to sentencing policy may have long-term unanticipated consequences. Indeed, harsher sentencing may hinder progress towards the intended goal of decreasing crime, creating safer communities and maximizing justice to the state, victim and offender. Our model suggests that increased sentencing for an individual has negative effects that spread through social networks to affect families and whole communities. As a consequence, increased sentence lengths may create criminals from individuals who otherwise would have avoided criminal behaviour.

The plausibility of social transmission suggests that the incarceration epidemic might be ameliorated by reducing the spread of incarceration through contact networks. In a model of contagion, reducing the overall probability of transmission reduces the steady-state prevalence of the epidemic. Transmission probabilities can be reduced by decreasing the time-rate of transmission or shortening the infectious period. Our model provides a framework for assessing the possible long-term effects of policies targeted at reducing transmission probabilities, but it is critical to first determine empirically the effectiveness of various policies. To this end, studies of the effect of different sentence lengths, alternative punishments, targeted interventions and retraining/re-integration programmes on ‘transmissibility’ are urgently needed. These data, in conjunction with models of contagion, would provide a substantially improved framework for analysis of incarceration-related policies and their cascading effects.

## Endnotes

1 Simulation code is available at http://ndssl.vbi.vt.edu/synthetic-data/.

2 http://www.ssa.gov/oact/STATS/table4c6.html.

3 http://www.cdc.gov/nchs/data/nvsr/nvsr62/nvsr62_01.pdf.

4 http://www3.norc.org/gss+website/.

5 http://www.cpc.unc.edu/projects/addhealth; data not publicly available.

6 http://www.bjs.gov/index.cfm?ty=pbdetail&iid=2056.
